# Predictors of adherence to micronutrient supplementation before and during pregnancy in Vietnam

**DOI:** 10.1186/s12889-017-4379-4

**Published:** 2017-05-16

**Authors:** Ines Gonzalez-Casanova, Phuong Hong Nguyen, Melissa Fox Young, Kimberly B. Harding, Greg Reinhart, Hieu Nguyen, Meredith Nechitillo, Truong V Truong, Hoa Pham, Son Nguyen, Lynnette M. Neufeld, Reynaldo Martorell, Usha Ramakrishnan

**Affiliations:** 1 Hubert Department of Global Health, Rollins School of Public Health, Emory University, 1518 Clifton Road NE, Atlanta, GA 30322 USA; 2 Poverty, Health, and Nutrition Division, International Food Policy Research Institute, 2033 K St, NW, Washington, DC 20006-1002 USA; 3 Thai Nguyen University of Medicine and Pharmacy, 284 Luong Ngoc Quyen St, Thai Nguyen City, Vietnam; 4 The Micronutrient Initiative, 180 Elgin Street, Suite 1000, Ottawa, ON K2P 2K3 Canada; 5 The Mathile Institute, 241 Taylor St, Ste 300, Dayton, OH 45402 USA; 6 The Global Alliance for Improved Nutrition (GAIN), Rue de Vermont 37–39, CH-1202 Geneva, Switzerland

**Keywords:** Micronutrient supplementation, Adherence, Women of reproductive age, Prenatal supplementation, Maternal nutrition, Side effects, Vietnam

## Abstract

**Background:**

Poor adherence to micronutrient supplementation often limits the effectiveness of public health programs. While predictors of adherence to micronutrient supplementation during pregnancy are well documented, information on adherence to preconception supplements is scarce. The objective of this study was to describe the predictors of adherence to preconception and prenatal micronutrient supplementation among women participating in a randomized control trial in Vietnam.

**Methods:**

Adherence data were collected prospectively from a double blind randomized controlled trial in rural Vietnam. Five thousand eleven women of reproductive age were randomized to receive preconception supplements for weekly consumption containing either: Folic Acid, Iron and Folic Acid (IFA), or Multiple Micronutrients. Women who became pregnant received prenatal IFA supplements for daily consumption through delivery. Village health workers visited participants’ homes every two weeks to deliver supplements and record consumption and side effects. Multivariate logistic regression was used to assess individual, household, and programmatic predictors of supplement adherence.

**Results:**

Adherence was high with 78 and 82% of the women consuming more than 80% of the preconception and prenatal supplements, respectively. Women of minority ethnicity (OR = 0.78 95% CI = 0.67, 0.91) and farmers (OR = 0.71 95% CI = 0.58, 0.88) were less likely to consume >80% of the preconception supplements while socioeconomic status (SES) (OR = 2.71 highest vs. lowest quintile; 95% CI = 2.10, 3.52) was positively associated with >80% adherence in the entire preconception sample with available information (*n* = 4417). Women in their first pregnancy had lower prenatal adherence compared to multiparous women. At the programmatic level, each village health worker visit was associated with higher odds of >80% adherence by 3–5% before pregnancy and 18% during pregnancy.

**Conclusions:**

Key determinants of adherence included SES, ethnicity, occupation (farmer) and parity which may be helpful for targeting women for counseling on supplement adherence. Increased contact with village health workers was positively associated with adherence to micronutrient supplementation both before conception and during pregnancy indicating the need for resources to support community outreach to women of reproductive age.

**Trial registration:**

NCT01665378. Registered on August 12, 2012.

## Background

Maternal nutritional status during pregnancy is an important determinant of child health, development, and well-being [1, 2]. Among various strategies, micronutrient supplementation during pregnancy has been identified as an effective intervention to improve maternal nutrition and pregnancy outcomes [3]. Moreover, the WHO has also issued recommendations to provide weekly iron folic acid (IFA) supplements to women of reproductive age in populations with a high prevalence of anemia as a measure to promote adequate nutritional status. Though this intervention is not recommended specifically to improve pregnancy outcomes (due to lack of evidence), it is widely accepted that improving women’s health prior to pregnancy will help ensure positive outcomes for mother and child [4].

A key determinant of success of nutrition supplementation interventions is how well participants adhere to the supplement regimen [5]. Adherence to prenatal micronutrient supplements, especially daily IFA supplements, has been found to vary across populations, and studies have identified socio-demographic and programmatic factors as important predictors [6]. For example, maternal education, occupation, socioeconomic status, and previous experiences with anemia or undernutrition have been identified as associated with adherence to daily IFA supplements during pregnancy [5, 7–10]. Similarly, side effects or metallic aftertaste have also been reported as barriers to adherence [5, 11]. However, these data, especially in low- and middle- income countries, have often been obtained from cross-sectional studies and/or are self-reported [1, 3, 5, 10–12]. Further, data on adherence to micronutrient supplementation within the context of a supplementation intervention prior to pregnancy (even among women of reproductive age) are limited.

We conducted a large double-blind randomized controlled trial of preconception micronutrient supplementation aiming to improve maternal health and birth outcomes (PRECONCEPT) [13, 14]. We have previously reported the findings from a qualitative study of beliefs and attitudes related to adherence to preconception and prenatal micronutrient supplementation that was conducted in a sub-sample of pre-pregnant, pregnant, and postpartum participants in the PRECONCEPT study [15], and found that women reported that they were more likely to adhere to supplements during pregnancy, as they considered them necessary for their and their offspring’s well-being. Additionally, side effects and forgetfulness were identified as barriers to adherence both before and during pregnancy [15]. However, the generalizability of these qualitative data is limited, and we were unable to estimate the magnitude of the effect of different barriers or facilitators on supplement adherence in the entire study population. The objective of this paper is to present quantitative estimates of predictors of adherence to preconception and prenatal micronutrient supplementation based on prospective data that were collected from all study participants.

## Methods

### Sample and study design

This study included women of reproductive age who participated in PRECONCEPT; a large randomized controlled trial of the effect of preconception micronutrient supplementation on birth outcomes in Northern Vietnam [14]. As part of this trial, 5011 women of reproductive age were randomized to receive weekly pre-pregnancy supplements containing Multiple Micronutrients (MM), Iron and Folic Acid (IFA), or only Folic Acid (FA). The composition and packaging of the supplements has been described previously [13]. Supplements were packed following manufacturing standards and only identified by letters and colors to assure the blinding during the intervention. The FA supplement contained 2800 mcg of folic acid, the IFA supplement contained the same amount of folic acid plus 60 mg of ferrous iron, and the MM supplement included the same amount of iron and folic acid plus 13 other micronutrients based on current international recommendations (Table 1). Based on WHO recommendations, the preconception supplementation followed a weekly schedule (1 pill per week) [16] and the prenatal supplementation followed a daily schedule (1 pill per day) [17].

**Table 1:** The composition of iron folate and multiple micronutrient supplements

Ingredient	Pre-pregnancy (weekly)		
	FA	IFA	MM
Vitamin A (μg)			800
Vitamin D (IU)			600
Vitamin E (mg)			10
Vitamin C (mg)			70
Thiamine (mg)			1.4
Riboflavin (mg)			1.4
Niacin (mg)			18
Vitamin B_6_ (mg)			1.9
Vitamin B_12_ (μg)			2.6
Folic acid (μg)	2800	2800	2800
Iron (ferrous sulfate) (mg)		60	60
Zinc (sulfate) (mg)			15
Copper			2 (mg)
Selenium (μg)			65
Iodine (μg)			150

After randomization, village health workers visited the homes of the participants every two weeks to deliver the supplements (observing the consumption of one tablet and delivering another with instructions to take it the following week) and recorded the self-reported number of pills consumed, side effects, and information about last menstrual period. If a delay in menstruation of over a month was recorded, women were given a pregnancy test. Women who became pregnant were referred to the local health clinic for their prenatal visits and switched to daily supplementation with IFA (prenatal supplements) as per Vietnamese standard of care protocols. Although these were packaged and labelled as prenatal supplements, the size and appearance of these supplements were similar to those provided before conception. The village health workers continued to visit pregnant women every two weeks to deliver the prenatal supplements and record consumption and side effects.

The study was designed so that the village health worker visited each woman every two weeks for the duration of the study during both the preconception and prenatal period. If it was known that a woman would be away from home for any of the visits, extra pills were given in advance to ensure a consistent dose. On the weeks when a visit was not programmed, the village health worker called or texted the participant to remind her to take the dose. During preconception and prenatal visits, village health workers also provided basic educational messages, including the importance of supplements to prevent anemia and improve health of the mother and the baby. They recommended taking the supplements at a fixed time so that they would not forget, and when side effects were reported, they suggested taking supplements about 2 h after a meal or changing the time the supplement was consumed.

This study was approved by Thai Nguyen University of Pharmacy and Medicine and Emory University’s Institutional Review Boards. Participation in the study was voluntary. Women provided informed consent prior to enrollment and randomization. A small monetary compensation (2 USD) for time and travel was provided to participants for study visits to the commune health centers at baseline and during pregnancy.

### Supplement adherence

**Fig. 1:**
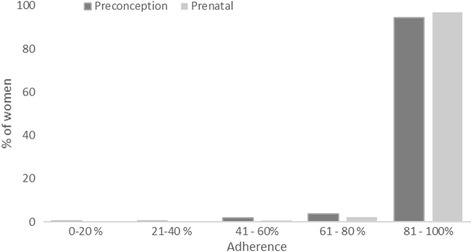
Distribution of preconception (*n* = 4417) and prenatal (*n* = 1183) adherence (% of supplements consumed) among Vietnamese women participating in PRECONCEPT

Adherence to the preconception supplement was calculated by dividing the total number of pills consumed (as registered by the village health worker) by the estimated number of pills each woman should have consumed. For women who did not become pregnant during the 2 years of follow-up, the denominator was calculated from date of the baseline interview to the last study day of preconception supplementation (in September 2014). For women who became pregnant during the study period, the denominator was calculated from the date of the baseline interview to the date when the pregnancy was detected. Adherence to the prenatal supplement was calculated by dividing the number of pills consumed (registered by the village health worker) by the number of pills they should have consumed calculated from the time the pregnancy was identified through delivery. Observations with missing information on dates or consumption were excluded from the analysis. Adherence was categorized into moderate adherence (≤80% of supplements consumed) or high adherence (>80% of supplements consumed) based on the high adherence to the supplement in the study population (Fig. 1).

### Predictors of adherence

We assessed whether factors at three different levels (individual, household, and programmatic) predicted adherence. At the individual level, we included maternal education (completed high school vs. less than high school), ethnicity (minority vs. Kinh majority), parity, occupation (farmer vs other), maternal underweight (defined as a Body Mass Index -BMI; below 18.5 kg/m^2^) and anemia (defined as hemoglobin concentration below 12 g/dl).

At the household level, we included a socioeconomic index (SES) and food insecurity. The SES index was constructed using principal components analysis considering land ownership, housing quality, access to services, and household assets. Household food security was assessed using the Household Food Insecurity Access Scale developed by the Food and Nutrition Technical Assistance Project [18].

The predictors at the programmatic level were PRECONCEPT supplementation group (MM or IFA vs. FA) and the number of visits from the village health workers.

An additional category of response to supplements included side effects or aftertaste reported by the participants, since these are a function of both individual responses and the type of supplement received.

### Statistical analysis

Bias due to missing data was assessed by comparing the characteristics of women included vs. those excluded due to missing information on the outcome variables using t-tests for continuous variables and Chi-Square tests for categorical variables. Differences in duration of supplementation, adherence to preconception or prenatal supplements, and reported side effect or aftertaste by preconception supplementation groups were also assessed using ANOVA or Chi-Square tests as appropriate. Multivariate logistic regression was used to identify associations between the predictors and the odds of high supplement adherence (>80%). For predictors of adherence to preconception supplementation, this analysis was conducted in the entire sample and then in the subsample of women who became pregnant. Generalized Estimating Equations (GEE) were used to account for the clustering at the VHW level (since women from the same village received the supplements and information from the same VHW). The use of GEE has been suggested as an effective approach to address data clustering [19]. We tested for multicollinearity among predictors of adherence to preconception and prenatal supplements in the final models. Additionally, we used Spearman ranked correlation test to assess the correlation between adherence before and during pregnancy.

## Results

A total of 4417 women were included in the preconception supplement adherence analysis and 1182 in the prenatal. The main reasons for exclusion from this analysis were incorrect dates or number of supplements recorded (*n* = 289), information missing on preconception or prenatal adherence (*n* = 275), and information missing on one or more predictors (*n* = 30).

Women participating in the study were on average 26 years old and most of them (~ 80%) worked as farmers (Table 2). Most women were on their second pregnancy but the proportion of first pregnancies was higher in the sample of women who did not become pregnant when compared to those who became pregnant (1.6% vs. 0.7%). There were no significant differences between women included in this analysis and those excluded due to missing information (results not shown).

**Table 2:** Characteristics of women participating in the preconception supplementation with multiple micronutrients on birth outcomes (PRECCONCEPT) trial in Vietnam by pregnancy

	All women (*n* = 4417)	Women who became pregnant (*n* = 1183)
Maternal Characteristics		
Age	26.2 ± 4.6	25.9 ± 4.4
Minority ethnicity	49.7	50.2
Farmer occupation	80.7	78.9
High school educated	22.5	20.9
Parity		
First pregnancy (%)	2.1	4.6
Weight (kg)	45.6 ± 5.6	45.8 ± 5.6
Height (cm)	152.5 ± 5.2	152.8 ± 5.1
BMI (kg/m^2^)	19.6 ± 2.1	19.6 ± 2.1
BMI < 18.5 kg/m^2^ (%)	31.6	32.0
Hemoglobin	12.9 ± 1.4	12.9 ± 1.3
Anemia (%)	19.9	19.9
Household Characteristics		
SES quintiles		
I (lowest)	20.1	18.9
II	20.0	20.8
III	19.9	20.2
IV	20.2	19.9
V (highest)	21.0	20.2
Food Insecure (%)	31.0	31.0
Programmatic Factors		
Supplementation group		
FA (%)	33.3	31.8
IFA (%)	33.4	33.1
MM (%)	33.3	35.1
Village health worker visits (#)		
Preconception	24.1 ± 13.8	14.6 ± 10.9
Pregnancy		11.9 ± 3.7

The proportion of women with high (> 80%) preconception and prenatal adherence was 78 and 81%, respectively. There were no significant differences in adherence by preconception supplementation group, but the duration of preconception supplementation was 2 weeks shorter in the MM group. A higher proportion of women in the MM group reported aftertaste and side effects, particularly nausea, vomiting, and headache. *“Bloody stools*” (dark-colored stools probably related to iron supplements) were more commonly reported in the MM and IFA groups compared to FA (Table 3). Among women who continued to take the preconception supplement during the first trimester of pregnancy (before they switched to the prenatal supplement), 5.8% reported any side effects, with nausea being the most common. A greater proportion of women taking MM (8.9%) or IFA (5.7%) during the first trimester reported side effects (especially nausea) compared to those who continued to take only FA (2.9%). The proportion of women who reported nausea or vomiting as a side effect of consuming preconception iron-containing supplements did not increase during the first trimester of pregnancy compared to the pre-pregnancy period. Among women who switched to the prenatal supplement during the first trimester, 10.7% reported side effects (data not shown).

**Table 3:** Duration, adherence, and side effects of preconception and prenatal supplements among 4417 women participating in PRECONCEPT a micronutrient supplementation trial in Vietnam

Preconception supplement^c^	MM (1469)	IFA (1489)	FA (1459)
Duration of supplementation (weeks)	54.0 ± 29.3	57.2 ± 28.7	57.1 ± 28.6 ^a^
Adherence (%)			
100%	17.2	14.6	14.7
80–99%	60.3	63.3	63.3
< 80%	22.5	22.1	21.9
Reported side effects (any; %)	31.0	19.2	15.8^a,b^
Fever (%)	2.8	2.8	2.3
Vomiting (%)	6.5	2.4	2.2^a^
Nausea (%)	20.2	9.3	7.3^a^
Bloody stool (dark color) (%)	5.0	4.7	2.7^a,b^
Diarrhea (%)	2.6	2.0	2.1
Headache (%)	13.3	9.9	8.2^a,b^
Vaginal Discharge (%)	1.6	1.5	1.4
Reported aftertaste (%)	13.1	8.0	6.6^a,b^
Prenatal Supplement^d,e^	MM (370)	IFA (404)	FA (409)^f^
Duration (weeks)	27.7 ± 8.0	27.5 ± 8.6	28.6 ± 7.9
Adherence (%)			
100%	19.5	23.2	17.9
80–99%	65.1	58.4	65.3
< 80%	18.0	18.5	19.0
Reported side effects (any; %)	12.4	15.3	13.9
Fever (%)	1.1	2.0	1.2
Vomiting (%)	4.6	5.7	4.7
Nausea (%)	7.6	5.9	9.1
Bloody stool (dark color) (%)	2.4	4.7	1.5
Diarrhea (%)	0.8	2.2	1.0
Headache (%)	4.3	3.7	4.4
Vaginal Discharge (%)	1.1	0.7	1.5
Reported aftertaste (%)	1.4	2.0	2.2

^a^MM group was significantly different from FA (*p* < 0.05)

^b^IFA was significantly different from FA (*p* < 0.05)

^c^PRECONCEPT was the supplement provided during the preconception supplementation intervention (MM: Multiple Micronutrients; IFA: Iron and Folic Acid; FA = Folic Acid)

^d^the prenatal supplement containing IFA

^e^Subsample of women who became pregnant

^f^The information is presented divided into preconception supplementation groups for consistency purposes, however all women received the same prenatal supplement (daily IFA) during pregnancy

Results from the multivariate logistic regression models that adjusted for all other predictors show that women of farmer occupation (OR = 0.78, 95% CI = 0.67, 0.91) and those of minority ethnicity (OR = 0.71, 95% CI = 0.58, 0.88) were less likely to consume >80% of the PRECONCEPT supplements (Table 4). Conversely, women of higher SES quintiles were more likely to reach this high adherence to preconception supplements (OR = 2.71 highest vs. lowest quintile; 95% CI = 2.10, 3.52). When the analysis was restricted to those who became pregnant, being underweight (OR = 0.65, 95% CI = 0.47, 0.91) or reporting aftertaste (OR = 0.53, 95% CI = 0.29, 0.97) were also inversely associated with high adherence in addition to the effects of belonging to a minority ethnic group (OR = 0.66, 95% CI = 0.47, 0.91). In this subsample, SES (OR = 3.90 highest vs. lowest quintile; 95% CI = 2.16, 7.05) was also positively associated with adherence, and food insecurity (OR = 0.69, 95% CI = 0.47, 0.91) was inversely associated.

**Table 4:** Predictors of adherence to preconception and prenatal supplements among 4417 Vietnamese women participating in PRECONCEPT

Odds of consuming >80% of supplements^a^	Preconception entire sample (*n* = 4417)	Preconception among women who became pregnant (*n* = 1183)	Prenatal (*n* = 1183)
Maternal Characteristics			
Age	1.00 (0.99, 1.02)	0.99 (0.96, 1.03)	1.02 (0.98, 1.06)
Minority ethnicity	0.78 (0.67, 0.91)*	0.66 (0.47, 0.91)*	0.84 (0.59, 1.19)
Farmer	0.71 (0.58, 0.88)*	1.27 (0.85, 1.91)	1.33 (0.91, 1.93)
Completed High school	0.96 (0.79, 1.17)	0.95 (0.64, 1.43)	0.99 (0.67, 1.45)
Underweight (BMI < 18.5 kg/m^2^)	0.93 (0.79, 1.09)	0.65 (0.47, 0.91)*	0.81 (0.59, 1.13)
Anemic (Hb <12 mg/dl)	1.04 (0.86, 1.25)	1.03 (0.69, 1.53)	1.41 (0.93, 2.14)
First pregnancy	1.26 (0.70, 2.27)	1.52 (0.65, 3.50)	0.54 (0.29, 0.99)*
Household characteristics			
SES index quintiles (vs. I Lowest)			
II	1.67 (1.34, 2.07)*	1.60 (1.02, 2.49)*	1.64 (0.97, 2.77)
III	2.00 (1.59, 2.52) *	1.75 (1.10, 2.80)*	1.53 (0.92, 2.55)
IV	2.17 (1.71, 2.76)*	3.09 (1.80, 5.31)*	1.51 (0.92, 2.50)
V	2.71 (2.10, 3.52) *	3.90 (2.16, 7.05)*	1.49 (0.91 2.44)
Food Insecure	0.88 (0.74, 1.04)	0.69 (0.49, 0.97)*	0.84 (0.59, 1.19)
Programmatic Factors			
Supplementation group (vs. FA)			
MM	0.98 (0.81, 1.17)	1.03 (0.69, 1.52)	1.10 (0.74, 1.62)
IFA	0.99 (0.83 1.19)	1.22 (0.83, 1.80)	0.90 (0.63, 1.30)
Village health worker visits	1.03 (1.02, 1.04)*	1.05 (1.01, 1.10)*	1.18 (1.13, 1.23)*
Response to supplements			
Aftertaste	1.05 (0.81, 1.36)	0.53 (0.29, 0.97)*	1.00 (0.52, 1.90)
Side effects	0.96 (0.80, 1.16)	1.40 (0.89, 2.20)	0.92 (0.61, 1.38)

^a^Odds ratios were estimated using Multivariate Logistic Regression to adjust for all other predictors in the table. *p < 0.05

In the case of the prenatal supplements, women on their first pregnancy were less likely to have high adherence to the prenatal supplement. At the programmatic level, a higher number of village health worker visits increased the odds of having high adherence to both the preconception (OR = 1.03 95% CI = 1.02, 1.04) and prenatal supplements (OR = 1.18 CI 95% = 1.13, 1.23). Adherence to preconception supplements was also positively correlated with adherence to prenatal supplements (ρ = 0.15; *p* < 0.001). Reporting side effects and being anemic at baseline were not significantly associated with adherence to the supplements either preconception or during pregnancy (Table 4).

These associations were not modified by the use of GEE in the multivariate logistic models to account for the clustering at the village health worker level.

## Discussion

Adherence to preconception and prenatal supplementation was high among Vietnamese women participating in a micronutrient supplementation randomized controlled trial. Over 75% of these women consumed >80% of pills, which is substantially higher compared to previous studies [5, 7–9]. The qualitative study highlighted that women perceived micronutrient supplementation, especially with iron, as a need only during pregnancy, or as a curative treatment rather than a preventive measure [15]. This quantitative analysis also shows that adherence to supplementation was slightly higher during pregnancy. However, the high adherence to supplements before pregnancy suggests that despite the beliefs that were expressed in the qualitative study, women were willing to consume the supplements that were delivered to them for free within the context of a randomized controlled trial with frequent home visits by community health workers.

Women of lower socioeconomic status and those who belonged to a minority ethnicity were less likely to adhere to the preconception supplementation. Similarly, farmer occupation in the general study population, and underweight and food insecurity among those who became pregnant, were associated with lower adherence to the preconception supplement. It is possible that these groups faced more barriers such as lack of time or problems remembering and thus had more difficulties adhering to supplementation, especially to the weekly schedule. As part of the qualitative study, lack of time and bad memory were highlighted as important barriers; similarly, women reported more difficulty adhering to the weekly supplement compared to the daily scheme that was implemented during pregnancy [15]. Moreover, in the qualitative study, women who believed that the dosing schedule was very strict were less likely to take the supplement when they remembered.

Evidence suggests social status is positively associated with cognitive flexibility (defined as the ability to switch from one concept to the other and adapt to new stimuli or circumstances), which in turn improves memory [20–22]. It is possible that women belonging to minority groups or of lower socioeconomic status had a more rigid interpretation of the supplementation schedule and hence were more likely to miss a dose if they did not remember to take it at the exact time it was programmed. Conversely, there is evidence of better adherence to nutritional interventions among participants of higher socioeconomic status who often are more aware of the benefits and importance [23].

Previous studies have also reported lower adherence to micronutrient supplementation among minorities, low-income, and less-educated women [11, 12]. These results are particularly concerning because they suggest that these groups, which are at greatest need as they are more likely to suffer from micronutrient deficiencies, are receiving lower doses due to poor adherence. Consequently, it will be important for future interventions to consider and address barriers specifically affecting these groups from the design stage. Potential alternatives could include the use of a lower dose of daily supplements to address memory concerns, more intensive monitoring and engagement with minority groups, and dissemination or communication strategies targeting specific believes or concerns.

The number of home visits by the village health workers was an important predictor of high adherence for both the preconception and the prenatal supplement. The great impact of these visits is potentially explained by the fact that village health workers observed the intake of a supplement during each visit. However, the encouragement, counseling, and reminders that were provided during the visits could also have improved adherence to the supplement. Although PRECONCEPT was designed as an efficacy trial that aimed to maximize adherence to the supplements, these findings suggest that face to face monitoring may be an effective strategy to improve adherence to micronutrient supplementation, but will require resources and training of front line workers. Alternatively, sending text message was a less intensive option to promote adherence and could be used in future interventions, especially in settings like Vietnam where most women use cellphones.

The occurrence of side effects has been highlighted as a deterrent from adherence to micronutrient supplementation [5, 11, 24, 25]. It was reported also as an important barrier in the PRECONCEPT qualitative study [15]. However, we found that side effects were not significantly associated with supplement adherence either preconception or during pregnancy. Moreover, there were no differences in adherence by supplementation group even though women who received the MM supplements were more likely to report side effects (than those in the IFA or FA groups). The lack of association between side effects and adherence is consistent with the landmark prenatal iron adherence study by Galloway et al. [26] in five developing countries, where only 10% of women stopped taking iron supplements because of the side effects. In that study, the main barrier for supplement intake was inadequate supply. Similarly, supplementation trials in China [27] and Bangladesh [28], reported no association between side-effects and adherence when supply of the supplement and contact with health personnel was provided. In the PRECONCEPT study, women had a reliable supply of supplements through the village health worker visits. Moreover, it is possible that the intense monitoring and frequent contact with the study team motivated women to consume the supplement despite the side effects. In this sense, it is important to consider that this was an efficacy trial and adherence is probably higher than expected under normal circumstances, due to high support and free provision of the supplements. It is likely that Zinc was responsible for the higher nausea, vomiting, and headaches, and aftertaste reported by women in the MM group [29]. Contrary to the null association with side effects, aftertaste was inversely associated with adherence in the subsample of women who became pregnant, suggesting the palatability of the supplements may be another important factor to consider when designing interventions.

In contrast with the many predictors of preconception adherence, only parity was associated with prenatal adherence. Women in their first pregnancy were less likely to have high adherence to the supplement than those in their second pregnancy. This could be explained by higher awareness of the importance of prenatal supplementation among women in their second pregnancy. It is also possible that it was harder for women who were pregnant for the first time to adhere to the supplement due to increased challenges and adjustments to being pregnant. The difference in predictors of adherence before and during pregnancy was unexpected but consistent with the small correlation found between preconception and prenatal anemia. These findings further support the need to understand the determinants of adherence to supplementation during the preconception period. Moreover, these results suggest that some barriers for preconception supplementation in Vietnam are resolved or become less important when women become pregnant. This could be potentially through a shift towards a more supportive environment for pregnant women or due to greater interest from participants to adhere to the supplement, possibly because, as found in the qualitative study, women consider prenatal supplements more relevant for their pregnancy and their child’s well-being.

A noteworthy result was the short duration of prenatal supplementation among women who became pregnant. Even though women were actively monitored and periodically screened for pregnancy, the average duration of prenatal IFA was only 28 weeks. This short duration of prenatal supplementation was due to a lapse between the time the pregnancy was identified in the study and women’s first prenatal visit. This finding further highlights the potential importance of preconception supplementation to help ensure adequate nutritional status during the early stages of pregnancy before pregnancy is confirmed and supplementation begins.

A limitation of this study is the missing information on adherence from approximately 10% of women. However, these women were not significantly different from those with adherence information available on basic socio-demographic characteristics. Similarly, as mentioned before, results from this trial portray adherence in a controlled and highly monitored environment, hence they may not be generalizable to effectiveness trials or program settings.

Conversely, important strengths of the study are the large sample size, that the information on supplement consumption and side effects was collected prospectively, and that there is information on adherence to supplementation from the preconception and prenatal periods. This is a unique study assessing predictors of adherence to preconception micronutrient supplementation in a large sample of women who expressed intent to become pregnant. Furthermore, information is available from the same women before and during pregnancy, which allowed us to study differences in the predictors of adherence from these two stages. Results from this analysis are useful to improve our understanding of the determinants of adherence to preconception and prenatal supplementation and can potentially lead to improvements in the design and targeting of strategies to increase adherence to micronutrient supplementation interventions.

## Conclusions

Adherence to micronutrient supplementation was high in this highly controlled randomized trial of preconception supplementation with multiple micronutrients. However, women at higher risk of nutritional deficiencies, such as those who belonged to a minority ethnicity, of farmer occupation, or those with low BMI, were less likely to have high adherence to the supplements before pregnancy. Future trials or programs should consider potential barriers that particularly affect these groups. Similarly, the ongoing contact with the participants and the bi-weekly visits by the village health workers seem to have had an important positive impact on supplement adherence before and during pregnancy. These are potential options to improve supplement adherence when enough economic resources are available.

## Abbreviations

BMI: Body mass index
FA: Folic acid
IFA: Iron and folic acid
MM: Multiple micronutrients
PRECONCEPT: Name of the preconception supplementation trial
SES: Socioeconomic status
